# PILOT TESTING OF NEWLY DEVELOPED OPEN EDUCATIONAL RESOURCES FOR AN AGING AND MENTAL HEALTH COURSE

**DOI:** 10.1093/geroni/igad104.1080

**Published:** 2023-12-21

**Authors:** Jocelyn McGee, Madison Ambrose, Sinai Wood, Chris Zakrzewski

**Affiliations:** Baylor University, Waco, Texas, United States; Baylor University, Waco, Texas, United States; Baylor University, Waco, Texas, United States; Baylor University, Waco, Texas, United States

## Abstract

The cost of secondary-level education is higher than ever in the United States. With the costs of tuition, housing, meal plans, and other necessary expenses, economically vulnerable students have difficulty locating affordable textbook options for their courses. In addition to finding affordable options, it is challenging to locate low-cost and credible up-to-date resources that best prepare students for the fields they wish to pursue after graduation. Furthermore, within these resources, the perspectives of diverse and underrepresented scholars are often overlooked. With an ever-increasing and diversifying United States, these areas need to be addressed within gerontological education to provide the best care for older populations. In this presentation, we detail our process of developing open education resources (OER) for an Aging and Mental Health course from a positive psychology framework integrating diverse perspectives and state of the art research, results from our first pilot test of the materials with a group of 25 students, and how we are going about adapting this course into an interactive Pressbook which will be available in the future at no-cost to the field.

